# Clinical Impact of the Incision of the Capsule Floor During Generator Replacement on Cardiac Implantable Electronic Device Infection Risk: A Single‐Center Experience

**DOI:** 10.1111/jce.16695

**Published:** 2025-04-21

**Authors:** Umut Celikyurt, Burak Acar, Hacer Dogan, Ipek Celikyurt, Kaan Hanci, Ozlem Guler, Aysen Agacdiken, Ahmet Vural

**Affiliations:** ^1^ Arrhythmia, Electrophysiology, Pacemaker Research and Management Center, Department of Cardiology Kocaeli University Medical Faculty Kocaeli Turkey; ^2^ Department of Pharmacology Kocaeli University Medical Faculty Kocaeli Turkey; ^3^ Department of Infectious Dieaseses Kocaeli University Medical Faculty Kocaeli Turkey

**Keywords:** cardiac device infection, fibrous capsule, generator replacement

## Abstract

**Introduction:**

The fibrous capsule around cardiac implantable electronic device (CIED) generators represents avascular tissue that could be colonized and provides the nidus for latent infection. The purpose of the study is to evaluate the effects of incision of the capsule floor at the lower and/or medial part at the time of generator replacement on the CIED infection and hematoma formation.

**Methods:**

This observational study with retrospective analysis of prospectively collected data included patients who underwent CIED generator replacement between January 2013 and January 2024. A total of 1059 consecutive patients were compared according to the incision of the capsule floor at the lower and/or medial part: 448 patients without (group 1) and 611 patients with an incision on the capsule floor (group 2).

**Results:**

Fifteen patients with CIED infection after generator replacement were identified. There were no significant differences between the two groups, except for a higher percentage of patients with number of previous procedures on pocket ≥ 2 (35% vs. 19.6%, *p* < 0.001), and NOAC use (10.6% vs. 6.7%, *p* = 0.027) in group 2. There was a lower infection rate in group 2 compared to group 1 (0.7% vs. 2.5%, *p* = 0.014). In multivariate analysis, independent predictors of CIED infection after generator replacement were replacement without an incision of the capsule floor (OR 4.384, 95% CI [1.355–14.189]; *p* = 0.014), and age< 65 years (OR 3.259, 95% CI [1.133–9.378]; *p* = 0.028).

**Conclusion:**

Generator replacement without incision of the capsule floor during generator replacement was associated with increased CIED infection risk. To minimize CIED infection risk, capsule floor incision could be considered during generator replacement.

## 1 Introduction

Over the past decades, the use of cardiac implantable electronic devices (CIED) has increased significantly. Generator changes, primarily due to battery depletion has increased in parallel to new implants [1]. Infection rates after CIED generator replacement are found to be significantly higher after device replacement procedures than after first implantation [2].

Although various risk factors for CIED infections after generator replacement, and prophylactic measures to reduce infection rates identified, procedure‐related interventions remain to be explored. Pocket‐based interventions to reduce CIED infections during generator replacement have been investigated before. The effects of capsulectomy at the time of generator replacement on CIED infection risk has been studied in recent years [1, 2, 3]. It has been shown that capsulectomy should not be performed routinely at re‐interventions, as it is associated with extended procedure length, pocket bleeding, and hematoma without improved infection rate [4, 5, 6]. However, incision on the capsule floor at the lower and/or medial part, without damaging the integrity of the capsule during generator replacement has not been studied yet. In this study, we aimed to present our experience, in terms of the effects of incision of the capsule floor at the lower and/or medial part at the time of generator replacement on the CIED infection and hematoma formation.

## 2 Methods

### 2.1 Patients and Study Protocol

This was a single‐center, observational study with retrospective analysis of prospectively collected data in patients who underwent CIED generator replacement. Consecutive patients who underwent generator replacement with or without capsule floor incision between January 2013 to January 2024 were included in the analysis. Written informed consent was obtained from all patients. The study was approved by the Local Ethical Committee.

The following demographic characteristics were collected: age, sex, and body mass index. Past medical history included coronary heart disease, hypertension, diabetes mellitus, chronic kidney disease, chronic heart failure, atrial fibrillation, and mechanical heart valve. Procedure‐related factors were as follows: CIED type, number of leads, procedure duration, incision of the capsule floor, number of prior procedures, temporary pacing, and postoperative hematoma. Baseline clinical and demographic information including medications, biochemical parameters, and international normalized ratio (INR) was collected before the procedure and recorded.

### 2.2 Procedure Details

Cardiac implantable electronic device generator replacements were performed in a dedicated electrophysiology laboratory under conscious sedation. All patients were brought to the laboratory in the fasting state, and after prophylactic intravenous antibiotics (cefazolin or vancomycin according to the operator's choice) were administered 30–60 min before the procedure [1, 7]. Generator replacement procedures were performed by the same 3 experienced operators (U.C., A.A., and A.V.). Povidone‐iodine antiseptic was used to clean the skin at the beginning of the procedure. After removing the existing generator from the pocket, a 2–3 cm length, and 1–2 cm deep incision was made by dissection scissors on the lower and/or medial floor portion of the capsule in patients who underwent generator replacement between February 2019 and January 2024. Pockets were not irrigated with antibiotic or saline solution in all procedures.

The procedure was performed on continued therapeutic anticoagulation with an international normalized ratio (INR) between 2 and 3 in patients taking warfarin. If INR was below 2 before the procedure, enoxaparin was started and continued until INR increases above 2. In patients with an INR value above 3, we interrupt the anticoagulation and wait until INR falls under 3 to perform the procedure. In patients receiving Non‐vitamin‐K antagonist oral anticoagulants (NOACs), the timing of intake of last NOAC before the procedure and restarting after the procedure were managed according to European Heart Rhythm Association practical guide [8]. Antibacterial envelopes were not available in our country, therefore none of the patients in our study received antibacterial envelopes.

### 2.3 Patient Follow‐Up

All patients were closely monitored for the development of fever or local signs of wound infection or hematoma during hospital stay. All patients were discharged the next day. No prophylactic antibiotics were administered routinely after the procedure. Upon leaving the hospital, patients and their families were advised to continue local wound care at home, and watch for signs of infection. Patients were instructed to seek prompt medical attention upon developing fever/chills or local signs of wound infection, regarding swelling, tenderness, redness, bruising, and wound dehiscence. Ten days after discharge the patients were advised to have the wound inspected by their physician or to visit our pacemaker clinic. All patients were followed every 6 months.

Cardiac implantable electronic device infection was defined as a fever > 38.0°C with one or more signs of wound inflammation, such as redness, swelling, tenderness, or purulent discharge. Hematoma was defined as a palpable swelling with fluctuance that extended beyond the device margin. Patients with CIED infection were admitted to our center. All patients with CIED infecton underwent extraction. Culture samples were obtained from the blood during hospitalization and from the pocket and from the lead during extraction.

### 2.4 Statistical Analysis

SPSS 20.0 Statistical Package Program for Windows (SPSS Inc. IL, USA) was used for statistical analysis. Patients were divided into two groups according to the capsule floor incision. Patient characteristics were compared between these two groups using a *t*‐test or *χ* ^2^ test, as appropriate. Univariate logistic regression analysis was used to evaluate variables associated with CIED infection after generator replacement. Variables with *p* < 0.10 in the univariate analysis were further entered into a multivariate logistic regression analysis to determine the independent predictors of CIED infection after generator replacement. The results of the regression analyses were presented as odds ratios (OR) and 95% confidence intervals (CI). A *p*‐value < 0.05 was considered statistically significant.

## 3 Results

**Figure 1:**
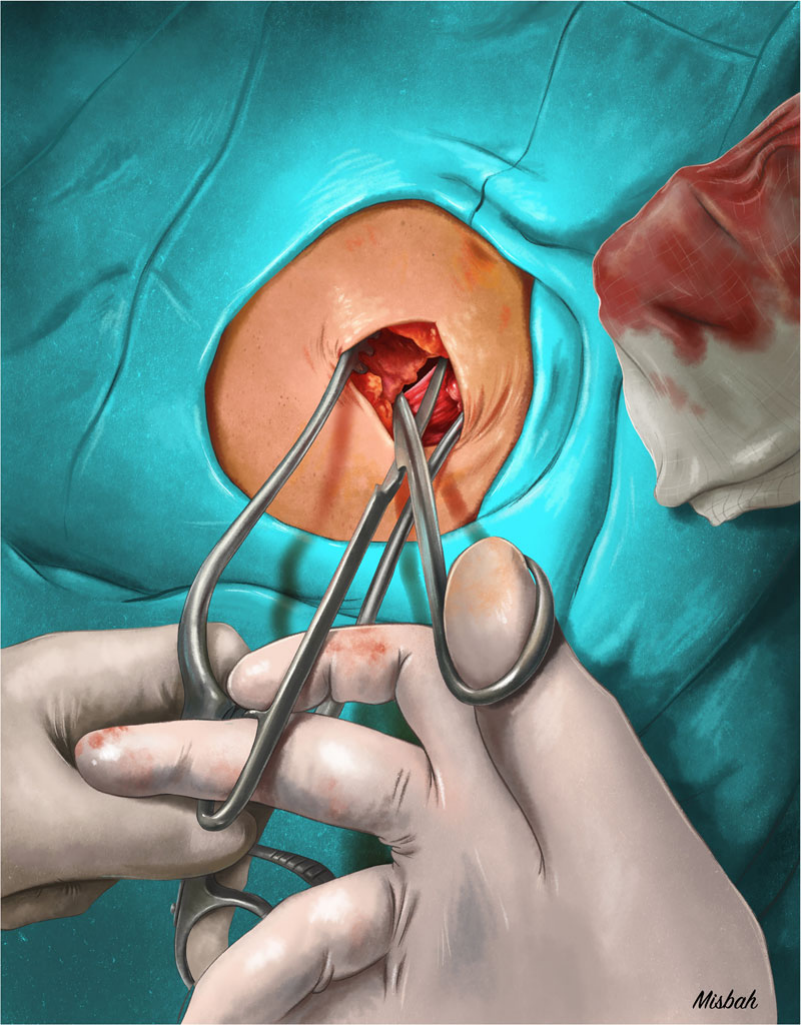
Illustration showing the incision of the capsule floor at the lower and/or medial part at the time of generator replacement.

A total of 1059 consecutive patients who underwent generator replacement at our tertiary university clinic between January 2013 and January 2024 were included in the study. Follow‐up data were available for all patients. The mean follow‐up was 32.7 ± 22.7 months. Patients were compared according to the incision on the capsule floor: 448 patients without (group 1) and 611 patients with an incision on the capsule floor (group 2) (Figure 1). Fifteen patients with CIED infection after generator replacement were identified.

Baseline characteristics of patients with and without capsule floor incision are presented in Table 1. There were no statistically significant differences between groups, except for a higher percentage of patients with number of previous procedures on pocket ≥ 2 (35% vs. 19.6%, *p* < 0.001), and NOAC use (10.6% vs. 6.7%, *p* = 0.027) in group 2. There was a lower infection rate in group 2 compared to group 1 (0.7% vs. 2.5%, *p* = 0.014) (Table 2).

**Table 1:** Baseline characteristics.

Patient characteristics	Patients without incision (*n* = 448)	Patients with incision (*n* = 611)	*p*‐value
Age, years	70.6 ± 13.7	69.6 ± 12.9	0.246
Female	177 (39.5)	252 (41.2)	0.570
BMI < 25 kg/m^2^	161 (35.9)	243 (39.8)	0.205
Hypertension	307 (68.5)	421 (68.9)	0.896
Diabetes mellitus	101 (22.5)	158 (25.9)	0.215
Coronary artery disease	141 (31.5)	214 (35.0)	0.227
History of heart failure	274 (61.2)	408 (66.8)	0.059
Atrial fibrillation	82 (18.3)	127 (20.8)	0.317
Mechanical heart valve	22 (4.9)	36 (5.9)	0.489
Chronic kidney disease	73 (16.3)	90 (14.7)	0.486
Hemodialysis dependency	13 (2.9)	9 (1.5)	0.107
LVEF, %	38 ± 19	37 ± 17	0.544
Device type			0.077
Pacemaker	180 (40.2)	204 (33.4)	
ICD	95 (21.2)	128 (20.9)	
CRT‐D	173 (38.6)	279 (45.7)	
Duration of procedure, min	29.5 ± 5.6	30.2 ± 6.4	0.074
Number of leads ≥ 3	180 (40.2)	276 (45.1)	0.060
Temporary pacemaker	31 (6.9)	55 (9.0)	0.221
Number of previous procedures on pocket ≥ 2	88 (19.6)	214 (35.0)	< 0.001
VKA	68 (15.2)	101 (16.5)	0.550
NOAC	30 (6.7)	65 (10.6)	0.027
Antiaggregants	248 (55.4)	320 (52.4)	0.336
INR	1.2 ± 0.4	1.2 ± 0.5	0.355
Bridging	19 (4.2)	18 (2.9)	0.257
Follow‐up	45.4 ± 26.8	24.6 ± 14.9	< 0.001

*Note:* Values are mean ± SD or *n* (%).

Abbreviations: BMI, body mass index; CRT‐D, cardiac resynchronization therapy–defibrillator; ICD, implantable cardioverter defibrillator; INR, international normalized ratio; LVEF, left ventricular ejection fraction; NOAC, non‐vitamin K antagonist oral anticoagulant; VKA, vitamin K antagonist.

**Table 2:** Differences in outcomes between the groups.

Patient characteristics	Patients without incision (*n* = 448)	Patients with incision (*n* = 611)	*p*‐value
Hematoma	12 (2.7)	17 (2.8)	0.919
Infection	11 (2.5)	4 (0.7)	0.014

*Note:* Values are *n* (%).

An organism was isolated from the pocket and/or blood in 60% of cases (9 of 15). Infection was diagnosed in six patients according to wound dehiscence and skin erosion with externalization of the generator or leads and purulent drainage from pocket, and fistula formation without positive blood cultures (Table 3). Infection rates were not significantly different between the operators (7/425 (1.6%) U.C.; 5/351 (1.4%) A.A.; 3/283, (1.1%) A.V.; *p* = 0.552).

**Table 3:** Patients with CIED infection details.

Patient no.	Age, years	Sex	Microorganism identified	Microorganism source	Time to infection (days post device replacement)	Incision of capsule floor
1	54	M	None	N/A	128	No
2	34	M	None	N/A	61	No
3	67	M	*Enterococcus faecalis*	pocket	53	No
4	48	F	*Staphylococcus epidermidis*	pocket	150	No
5	70	M	*Staphylococcus epidermidis*	Pocket and bloodstream	36	No
6	62	M	None	N/A	49	No
7	69	M	*Staphylococcus epidermidis*	bloodstream	82	No
8	79	F	*Serratia marcescens*	pocket	102	No
9	51	M	None	N/A	183	No
10	50	F	*Staphylococcus aureus*	pocket	390	No
11	57	F	*Staphylococcus aureus*	pocket	30	No
12	64	M	*Staphylococcus aureus*	bloodstream	332	Yes
13	59	M	*Staphylococcus epidermidis*	pocket	113	Yes
14	70	M	None	N/A	55	Yes
15	79	F	None	N/A	138	Yes

Abbreviations: CIED, Cardiac implantable electronic device; N/A, not available.

The possible confounder factors for CIED infection after generator replacement were first assessed in univariate logistic regression analysis. (Table 4) The parameters with *p* < 0.10 were further performed in multivariate analysis. (Table 4) In multivariate analysis, independent predictors of CIED infection after generator replacement were replacement without an incision of the capsule floor (OR 4.384, 95% CI [1.355–14.189]; *p* = 0.014), and age < 65 years (OR 3.259, 95% CI [1.133–9.378]; *p* = 0.028).

**Table 4:** Univariate and multivariate predictors of pocket infection after generator replacement.

Variables	HR	95% CI	*p*‐value	HR	95% CI	*p*‐value
Number of previous procedures on pocket ≥ 2	0.343	0.123–0.954	0.040	0.401	0.131–1.222	0.108
Number of leads ≥ 3	0.275	0.087–0.869	0.028	0.353	0.103–1.212	0.098
History of heart failure	0.660	0.209–2.086	0.479			
Age < 65	3.651	1.289–10.347	0.015	3.259	1.133–9.378	0.028
No incision of capsule floor	3.820	1.208–12.075	0.022	4.384	1.355–14.189	0.014
Duration of procedure	0.992	0.913–1.077	0.846			
NOAC	0.636	0.141–2.860	0.555			

Abbreviations: CI, confidence interval; HR, hazard ratio; NOAC, non‐vitamin K antagonist oral anticoagulant.

## 4 Discussion

The main findings of this study are as follows. (1) Incision of capsule floor resulted in a significantly lower CIED infection rates after generator replacement. (2) Incision of the capsule floor does not increase bleeding risk and hematoma formation.

Pocket‐based interventions to reduce CIED infections during generator replacement have been investigated before. Some researchers suggest that the capsule represents an avascular tissue and may promote bacterial colonization, and latent infection [2, 5, 9]. It has been suggested that capsulectomy results in revascularization of the pocket and allows better antibiotic penetration. The prospective randomized single‐center MAKE IT CLEAN trial evaluated the impact of excision of the fibrous capsule at the time of generator replacement on infection reduction [1]. The floor and roof of the fibrous capsule were removed in pocket revision group. They revealed that procedure times were significantly longer in patients undergoing generator replacement in the pocket revision group. In our study, procedure duration was slightly longer in group 2, however it was not statistically significant. Incision of the capsule floor could be easily performed without prolonging the total procedure duration significantly. It is known that prolonged procedure duration is associated with a higher risk of infection [5]. New techniques to minimize the duration of an open pocket are required. Therefore, our approach could feasibly be applied routinely to reduce CIED infection risk without prolonging the procedure duration during generator replacement.

Lakkireddy et al. also reported increased bleeding rates in patients who underwent pocket revision [1]. In our study, there was no significant difference in baseline INR or use of antiplatelet therapy between the groups. NOAC use is significantly higher in group 2, which may be related to the steeply increased use of NOACs in recent years. However, it was not found to be a predictor of infection after generator replacement in univariate analysis. There was no difference in hematoma formation between the groups. The reason may be related to the undamaged pocket integrity, as we did not removed any part of the capsule. Furthermore, incision of the capsule floor probably helps in revascularization of the pocket, and better antibiotic penetration, and prevents nidus formation for future infections.

It is known that inadvertent damage to the leads within the pocket is a potential complication during generator replacement [10]. It has been also reported that implantable cardioverter‐defibrillator generator replacement is associated with an increased risk of lead alert compared to age‐matched leads without replacement, and it is recommended to avoid excessive force during lead dissection and minimize manipulation of the lead [11]. Compared to additional capsulectomy during generator replacement, incision of the capsule floor does not require dissection to free the leads from its fibrous attachments. There was no incidence of lead damage following generator replacement in our study. Incision of the capsule floor to reduce CIED infection is a safe technique without lead damage risk during generator replacement.

We observed that age < 65 was an independent risk factor associated with CIED infection after generator replacement. It has been reported that an inverse relationship exists between increasing age and the risk of infection. The presence of less firm subcutaneous connective tissue, and a less aggressive immune response against low virulence bacteria with increasing age have been proposed as underlying causes [12].

Previous studies suggested that number of prior CIED procedures has been associated with CIED infection [5, 13, 14]. The procedures included (generator replacements, reinterventions, and upgrades) in the studies may affect the results. Higher number of leads and previous procedures on pocket were not predictive of CIED infection after generator replacement in our study. This suggests that the replacement procedure, rather than the previous procedures and number of leads, was responsible for the infection after generator replacement.

World Health Organization recommended alcohol‐based antiseptic solutions for surgical skin antisepsis [14]. However, alcohol‐based antiseptics is not available in our hospital. Povidone‐iodine solution was used for skin disinfection in all patients, and the study results are not affected by the antiseptic agent used. The use of the same antiseptic and antibiotic prophylaxis in all patients allows us to identify other predictors of CIED infection after generator replacement.

### 4.1 Study Limitations

Our study has several strengths, including a large number of patients, and the relatively long follow‐up period. However, we acknowledged that this study still had limitations. This is a single‐center, nonrandomized study, and inherent limitations associated with this kind of design should be considered. It is possible that though some fibrous capsule was removed and it may not have been documented in the procedure note. While a large number of patients are included, concern regarding temporal bias could be raised. It is also important to note that we report our experience in a high‐volume device implantation center. Cardiac implantable electronic device infection rates after generator replacement could vary among CIED implantation centers.

## 5 Conclusion

Independent predictors of CIED infection after generator replacement include replacement without an incision of the capsule floor and age < 65. Our results suggest that capsule floor incision could be considered during generator replacement to minimize CIED infection risk. Further randomized controlled trials are needed to confirm our results.

## Conflicts of Interest

The authors declare no conflicts of interest.

## Data Availability

The data that support the findings of this study are available on request from the corresponding author. The data are not publicly available due to privacy or ethical restrictions.
